# The Responsibilities of Physicians Who Attend Patients Affected with Contagious or Infectious Diseases

**Published:** 1890-11

**Authors:** 


### THE RESPONSIBILITIES OF PHYSICIANS WHO ATTEND PATIENTS AFFECTED WITH CONTAGIOUS OR INFECTIOUS DISEASES.

---

Ever and anon instances present themselves to us where physicians, who are in attendance upon dangerous contagious and infectious diseases, either from ignorance or indifference, partially or totally, disregard those simple precautions which are absolutely necessary to the safety of communities, to say nothing of individuals and families. Cases of scarlet fever are treated without any attempt at isolation or disinfection of the patient, and subsequent thorough disinfection and fumigation of infected clothing, bedding, apartments, etc., and not infrequently the doctor quiets the apprehensions of the family by pronouncing the case "scarlatina" instead of scarlet fever. There is a prevalent idea among the laity that scarlatina is a very much milder disease than scarlet fever, which it is the duty of every true physician to contradict. The people should know that they are synonymous terms. They should be taught that the most violent cases may be contracted from the mildest.

So are many cases of typhoid fever treated as simple continued fevers without the slightest attempt at disinfection of the dejecta, and very often in typical typhoid cases there is only a quasi attempt at sterilization of the stools before they are dumped into privies, or cess-pools or buried in the soil adjacent to the well, from which water for drinking and domestic purposes is obtained. Thus one case may contaminate the water supply of an entire community. The physician in attendance who has failed to do his whole duty is, of course, indirectly responsible for all the suffering and deaths resulting.

In country districts, and in the smaller towns there are no laws to compel the carrying out of essential prophylactic measures, but every physician is amenable to that higher and unwritten law—the good of humanity—and must hold himself criminally negligent when he fails to practice every precaution to prevent the spread of disease.

---

### ITEMS.

DR. W. B. PRATHER has removed from Florence, Ga., to Seale, Ala.

---

Dr. CHAS. O. BROCK has changed his post-office from Jefferson to Mozelle, Ga.

---

Dr. I. S. STONE has removed from Lincoln, Va., to Washington, D. C., No. 1309 H. Street, N. W.

---

### MODERN METHODS OF MANAGING LINGERING LABOR.

---

The discussion upon the above important subject by the members of the Obstetric section of the British Medical Association, led by Dr. Playfair, at the recent meeting in Birmingham, emphasized many points in modern obstetric practice at variance with methods of procedure in vogue a few years since, and still upheld by that class of obstetricians who hide a multitude of faults under the pretext that meddlesome midwifery is bad. The general consensus of opinion was that lingering first stages call for appropriate treatment just as do lingering second and third stages. That chloral, opium and gelsemium were preferable to anæsthesia in the first stage with rigid os and insufficient pains. That gentle friction or pressure over the uterus to promote contractions, or these measures failing, delivery with the forceps in prolonged second stages were called for. That ergot was unsafe at any period of labor, and that there should be a distinct dis-association in the minds of obstetricians of high and low forceps operations.

---

### A PLEA FOR CONSERVATISM IN MINOR GYNECOLOGY.

That there is too much minor gynecology at the present day is a very common opinion, especially among those who see much serious pelvic disease. A plea for greater conservatism was lately made by Dr. Joseph Price before the Philadelphia County Medical Society, and was sympathetically received by the gentlemen taking part in the discussion.

Many cases of major pelvic trouble are due to minor operations, which have become the fashion to such an extent that they are often looked upon as routine treatment by a large class of practitioners of more or less limited gynecological training. But diagnosis in the pelvis is difficult; dormant or unrecognized pelvic inflammation is not infrequently lighted up by an apparently trivial procedure and the foundation laid for a future laparotomy.

Emmet himself has called attention to the dangers of his cervical operation except in carefully selected cases. Many patients are left in a worse condition after the repair of the cervix than they were in before. It should not be undertaken except in case of absolute necessity until the absence of any pre-existing pelvic disease has been ascertained.

Another favorite operation which is much abused is the forcible dilatation of the cervix. Dysmenorrhœa is not relieved by this process unless it be due to actual stenosis of the os or cervix, but many women with what might be considereda stenos is of the os have no dysmenorrhœa. A very small opening is all that is required for menstrual drainage. It is therefore necessary to make a careful study of all symptoms before concluding that the trouble is due to obstruction to the flow. Although relief is often obtained, it should be borne in mind that forcible dilatation is a distinct traumatism, and as such is never entirely free from danger.

The uterine sound is responsible for much inflammatory pelvic trouble, especially since it has become a common instrument for diagnosis and is a particular favorite of the less skilful examiner, although nearly discarded by many men of larger experience.

A strong objection to the use of electricity by the tyro is the mechanical disturbance due to the frequent and careless introduction of the sound and electrode. The curette also, although a valuable instrument, has been the indirect, as well as direct, source of a long list of victims. And finally the routine treatment by caustic intra-uterine injection has often hastened, if not caused, pelvic inflammatory trouble leading at last to abdominal section.—*Boston Medical & Surgical Journal, October 16, 1890.*

---

*The British Journal of Dermatology* quotes from a paper by M. Barthelemy, read at July meeting of the French Society of Dermatologists, on twenty-eight autopsies on syphilitic still-born infants. Most presented no lesion visible, either to the eye or microscope. They were killed by the syphilitic virus, without any alteration of the organs; just as may happen in the case of variola. In five cases there were gummata, affecting, in three cases, the liver alone, in one case the liver, tibias and clavicles, and once the thymus also. In eight other cases where no microscopic gummata were apparent, the liver, in five cases, presented a granular appearance, the microscope revealing the presence of miliary gummata of the liver and kidneys. No lesions of spleen were noticed, save that it was generally larger than normal.

The above Journal also mentions a case of Locomotor Ataxia of syphilitic origin, cured by "specific" treatment. Chancre, in '76, treated only locally. In '80 typical locomotor ataxia began, lancinating pains, ataxic gait, diplopia, plantar anæsthesia, abolition of tendon reflexes, etc., lasting nearly three years. After six weeks of "mixed treatment" most of the symptoms disappeared, and gradually every symptom disappeared, and patient has been well since '83, still taking an occasional course of potassium iodide.

---

### ELECTRICAL CATAPHORESIS IN TREATMENT OF SYPHILIS.

---

Dr. Ehrmann read a paper upon this subject at the last International Medical Congress, which is reported in Monatshefte für Praktische Dermatologie of September 15. He mentions as objections to inunction that you either get too much or too little mercurial absorption, and says we need a treatment which will bring the desired quantity of mercurial into the system without the prior production of a lesion, as in the injection method. Acting upon the principle of electrical osmosis, through a porous partition, from the anode to cathode, he made experiments and observed that the substance dissolved in the fluid (methyl-violet) was forced into follicles of the skin of the anode side but not into those on cathode side. Next, finding that he was able to introduce a given quantity of bichloride of mercury into the system, he treated syphilitic cases by this means, thirty-four in all. Results were almost uniformly good. Diarrhœa occurred in only two cases, and ptyalism in only one.

Of late he employs the baths (electrical) every day or every other day, using twelve grains bichloride of mercury, and a current of 200 milliamperes. Duration of bath half an hour, current being reversed after fifteen minutes, as follicles on anode side could receive no more, necessitating change of cathode into anode.

This application of electricity has been recommended as a means of causing bichloride solution to penetrate the hair follicles for the destruction of the fungus of that obstinate affection, ringworm of the scalp. But, altogether, electrical cataphoresis must yet be considered in the infancy of its application, and further trial is necessary to demonstrate its true value.

WARNER: SCIENTIFIC STUDY OF THE CONDITION OF CHILDREN IN SCHOOLS (*The Lancet*, April 5, 1890.)

In this paper the author considers the possible value of accurate observation of facts seen in children in schools as a means of aiding the solution of educational problems. There is, at present, but little accurate scientific observation of children in schools from the point of view of ascertaining their special adaptation to and requirements in education.

Looking upon a body of children as pupils in a school, we would wish to classify them according to their (1) development; (2) nutrition and physical health; (3) brain condition. The physical signs of these may be learned by any observer; but, like all new physical signs, they require some moderate degree of application to recognize them.

The effects of education and various modes of teaching upon the brains of the children can only be determined by actual observation in the schools. There is no body of facts founded upon extended observation of school-children, showing their condition and its bearing upon the adult population of the next decade.

What is wanted is a careful investigation, conducted by observation, of the children along definite lines, as well as by inquiry of the teachers. At present we have no standard to go by, no average of the conditions we desire to remove. The study of pathology and clinical diagnosis must precede scientific treatment, and we need some exact knowledge of the condition of samples of the school population before science can take its proper place in directing education, or give due service to the State by advice upon many of the practical problems put forward by the educationist for solution.

---

NEUMANN. "Bromoform in whooping-cough."—*Therap. Monatshefte*, July, 1890.

SINCE the beginning of 1889 the author has had under treatment 61 cases of whooping-cough, 25 of which were treated with bromoform, the rest with other drugs. The drug was given in drops suspended in syrup, and no unpleasant effects were observed, the dose being 3—5 drops frequently repeated. Neumann believes it to cut short the course of the disease, and it certainly seems efficacious in reducing the number of paroxyms.

---

NEUSSER.. "Potassium tellurate ($K_2$ Te $O_4$) in night sweats of phthisis." *Wiener klin Wochenschrift*, 1890, No. 23.

NEUSSER gave this salt in doses of .02 to .04 grammes in pill to phthisical patients. No alteration in the progress of the disease was noticed, but the night sweats were in a great many cases either stopped or materially lessened. After a week toleration became established and the doses had to be increased. Toxic symptoms, chiefly gastric dyspepsia, etc., occurred only after daily doses of .06 grammes. In all cases there was an intense odor of garlic in the breath.

---

### IN MEMORIAM.

ATLANTA, October 18, 1890.

WHEREAS, The students of the Southern Medical College and the members of the Southern Medical Society, of Atlanta, this day in mass meeting assembled to take action upon and draft such resolutions as may be proper on the death of two of our beloved professors, Drs. Wm. D. Bizzell and Robert C. Word, therefore be it

*Resolved*, That in the wisdom and all-seeing power of the Great Omnipotent, this time-honored institution has suffered an overwhelming and dire calamity, Atlanta has lost two of her most valued citizens, and their places in the ranks of the profession shall ever after be vacant. Be it also

*Resolved*, That in their death the public is bereft of two of her most noble and self-sacrificing benefactors, and that they shall be sadly mourned and missed on their daily missions of mercy and peace. Also, be it

*Resolved*, That to the families of our esteemed tutors we extend our deepest sympathies, and express the hope that the Supreme Physician of the universe may attend them in their sore bereavement, and administer such condolence as he alone can give. Be it also

*Resolved*, That a copy of these resolutions be sent to their respective families, also to the ATLANTA MEDICAL JOURNAL for publication.

Respectfully submitted,
C. E. JOHNSON, Chairman.
M. M. MCGEHEE,
C. H. FINDLEY, Committee.

---

### PRELIMINARY PROGRAMME OF THE SESSION OF THE SOUTHERN SURGICAL AND GYNECOLOGICAL ASSOCIATION TO BE HELD IN ATLANTA, GEORGIA, NOVEMBER 11TH, 12TH AND 13TH, 1890.

---

PRESIDENT.

GEORGE J. ENGELMANN, M. D., St. Louis Missouri.

VICE-PRESIDENTS.

B. E. HADRA, M. D., Galveston, Texas.
DUNCAN EVE, M. D., Nashville, Tennessee.

SECRETARY.

W. E. B. DAVIS, M. D., Birmingham, Alabama.

TREASURER.

HARDIN P. COCHRANE, Birmingham, Alabama.

JUDICIAL COUNCIL.

JOHN S. CAIN, M. D., Nashville, Tennessee.
WM. T. BRIGGS, M. D., Nashville, Tennessee.
HUNTER McGUIRE, M. D., Richmond, Virginia.
VIRGIL O. HARDON, M. D., Atlanta, Georgia.
BEDFORD BROWN, M. D., Alexandria, Virginia.

CHAIRMAN OF THE COMMITTEE OF ARRANGEMENTS.

VIRGIL O. HARDON, M. D., Atlanta, Georgia.

**Members of the Medical Profession are cordially invited to attend.**

### PAPERS TO BE READ.

(PARTIAL LIST.)

| | |
|---|---|
| The President's Annual Address. . . | GEORGE J. ENGELMANN, M. D., St. Louis, Mo. |
| How Shall We Treat our Cases of Pelvic Inflammation? . . . . . . | R. B. MAURY, M. D., Memphis, Tenn. |
| The General and Local Treatment of Gangrenous Diseases and Wounds, . . | BEDFORD BROWN, M. D., Alexandria, Va. |
| Further Study of the Direct and Reflex Effects of Lacerations of the Female Perineum, . . . . . . . | J. H. BLANKS, M. D., Nashville, Tenn. |
| Abdominal and Pelvic Surgery in America, | JOSEPH PRICE, M. D., Philadelphia, Pa |
| Intraligamentous Ovarian Cystoma, . | CORNELIUS KOLLOCK, M. D., Cheraw, S. C |
| Anatomy and Pathology of the Ileo-Cæcal Region, . . . . . . . | RICHARD DOUGLAS, M. D., Nashville, Tenn. |
| Wet Antiseptic Dressings in Hand Injuries, | WM. PERRIN NICOLSON, M D., Atlanta, Ga. |
| The Best Route to the Bladder in the Male for Disease or for Foreign Bodies, | HUNTER McGUIRE, M. D., Richmond, Va |
| Suprapubic Cystotomy in a Case of Enlarged Prostate, . . . . . . | WM. H. H. COBB, M. D., Goldsboro, N. C. |
| Indications for Cholecystotomy, . . | A. M. OWEN, M. D., Evansville, Ind. |
| Uterine Moles and Their Treatment, . | J. T. WILSON, M. D, Sherman, Tex. |
| Strictures of the Male Urethra, . . | W. F. WESTMORELAND, M. D. Atlanta, Ga. |
| Treatment of Urethral Strictures by Electricity, . . . . . . . | W. FRANK GLENN, M. D. Nashville, Tenn. |
| The Surgical Treatment of Empyema, . | J. A. GOGGANS, M. D. Alexander City, Ala. |

| | |
|---|---|
| Cases in Abdominal Surgery, . . . | I. S. Stone, M. D., Lincoln, Va. |
| Rectal Medication in Pelvic Troubles, . | W. Hampton Caldwell, Lexington, Ky. |
| Conservative Surgery in Injuries of the Foot, . . . . . . . . | J. T. Wilson, M. D., Sherman, Texas. |
| The Management of the Infantile Prepuce, | George Ben Johnston, Richmond, Va. |
| The Ultimate Results of Trachelorrhaphy . | Virgil O. Hardon, Atlanta, Ga. |
| Further Observations on the Dangers of Operative Delay in Prostatic Troubles, with Personal Experience, . . . | R. D. Webb, M. D., Birmingham, Ala. |
| Clinical History of the Epicystic Surgical Fistula, with Cases, . . . . | Jno. D. S. Davis, M. D., Birmingham, Ala. |
| Foreign Bodies in the Air Passages, with Report of Cases, . . . . . | John E. Pendleton, M. D., Hartford, Ky. |
| Cholecystotomy, . . . . . . | W. E. B. Davis, M. D., Birmingham, Ala. |
| Two Cases of Laparotomy for Intestinal Obstruction, . . . . . . . | J. T. Jelks, M. D, Hot Springs, Ark. |
| Is Gonorrhœa ever a Cause for Pelvic Inflammations? . . . . . | J. R. Buist, M. D., Nashville, Tenn. |
| (Title of paper not determined), . . . | W. O. Roberts, M. D., Louisville, Ky. |
| (Title of paper not determined), . . . | L. S. McMurtry, M. D., Louisville, Ky. |
| (Title of paper not determined), . . . | Wm. D. Haggard, M. D., Nashville, Tenn. |
| (Title of paper not determined), . . . | Hunter P. Cooper, M. D., Atlanta, Ga. |

George J. Engelmann, M. D.,

*President.*

W. E. B. Davis, M. D.,

*Secretary.*

